# Evaluation of Health Literacy Levels in Patients in the Emergency Department of a University Hospital: A Cross-Sectional Study †

**DOI:** 10.3390/healthcare14121665

**Published:** 2026-06-11

**Authors:** Gulsum Ozturk Emiral, Pakize Gozde Gok, Alaettin Unsal, Didem Arslantas, Engin Ozakin, Nurdan Acar

**Affiliations:** 1 Public Health Department, Faculty of Medicine, Ankara Yıldırım Beyazıt University, 06010 Ankara, Turkey; dr.gulsum.ozturk@gmail.com; 2 Emergency Department, Yunusemre State Hospital, 26190 Eskişehir, Turkey; 3 Public Health Department, Eskişehir Osmangazi University Hospital of Health, Clinical Practice and Research, 26040 Eskişehir, Turkey; 4 Emergency Department, Eskişehir Osmangazi University Hospital of Health, Clinical Practice and Research, 26040 Eskişehir, Turkeynurdanergun@gmail.com (N.A.)

**Keywords:** health literacy, healthcare utilization, patient education

## Abstract

**Aim**: This study aimed to assess the health literacy (HL) levels of patients visiting the emergency department of a university hospital and identify related factors. **Methods**: This cross-sectional study aimed to assess the health literacy levels of patients visiting the emergency department of a university hospital, and to identify related factors. The re-quired sample size was at least 384 individuals, assuming an inadequate HL level of 50%, with 95% confidence interval and 5% margin of error. Data were collected through a two-part questionnaire designed by the researchers. The first part covered the patients’ socio-demographic characteristics and details regarding their emergency department visits. Meanwhile, the second part included the widely used Chew’s short questions to assess inadequate HL. The analysis was conducted using IBM SPSS version 27.0. Descriptive sta-tistics, including frequency, percentage, and mean, were used to summarize the charac-teristics of the study group. The Chi-square test was applied for data analysis. **Results**: The study group included 58% (*n* = 250) female and 42% (*n* = 181) male. Their ages ranged from 18 to 64 years, with a mean (SD) of 29.6 (10.8) and a median of 26.0. In terms of HL levels, 197 individuals (45.7%) had inadequate HL. The frequency of inadequate HL was higher in individuals over the age of 40 years and those with an education level of ≤8 years (*p* < 0.05 for each). A total of 39.2% (*n* = 169) of the patients had visited the emergency department multiple times for their current complaints, whereas 243 participants (56.4%) visited the emergency department for a different reason within the past six months. **Conclusions**: In our study, four out of ten individuals had inadequate HL, and the frequency of repeated emergency department visits was quite high. No statistically significant association was found between emergency department usage characteristics and health literacy levels in the present sample, highlighting the need for larger longitudinal studies with adjusted analyses.

## 1. Introduction

Health literacy (HL), widely recognized as a key determinant of health outcomes, is defined as “the entire process of accessing, understanding, and processing essential information that enables individuals to make accurate health-related decisions [1,2].”Although HL is closely related to general literacy, it is not entirely equivalent to it. Research has shown that low HL is linked to reduced use of preventive health services, increased reliance on emergency care due to acute exacerbations of chronic conditions, high hospitalization rates and poor clinical outcomes, thus resulting in greater healthcare costs [3,4,5,6,7,8,9,10,11,12,13,14,15,16,17,18,19].

The American College of Emergency Physicians defines emergency healthcare services as “services provided to evaluate and/or treat any condition in which an individual with an average level of medical/health knowledge, who is not a healthcare professional, believes that immediate and unplanned medical care is necessary [20].” Although the concept of “average health knowledge” is not clearly defined, it is evident that low HL levels negatively affect the use of emergency services. The significance of HL is further heightened in high-pressure environments such as emergency departments, where rapid and complex decision-making is required and following medical instructions becomes even more challenging. Depending on the study type and assessment tools used, the prevalence of inadequate HL in emergency departments has been as high as 88% [21,22,23]. Research has shown that individuals with low HL are more likely to visit emergency departments for preventable reasons, have a higher risk of repeat visits, and incur greater healthcare costs [21,23,24].

The unnecessary use of emergency departments causes economic strain on the healthcare system and a psychological burden on healthcare professionals because of overcrowding. This can result in delays for patients who are in genuine need of emergency care and decline the overall quality of healthcare services. Identifying the reasons for unnecessary repeated emergency department use may contribute to reducing emergency department overcrowding and healthcare-related economic burden. To the best of our knowledge, there are limited studies evaluating health literacy levels among clinically stable adult patients presenting to emergency departments in Türkiye using validated health literacy screening tools. In addition, evidence regarding the relationship between sociodemographic characteristics, perceived income level, and health literacy in emergency department populations remains limited. The aim of this study was to evaluate health literacy levels among clinically stable adult triage category-3 patients aged 18–64 years presenting to the emergency department of a university hospital and to examine factors associated with inadequate health literacy in this specific patient population.

## 2. Materials and Methods

This descriptive study was conducted at the adult emergency department of a tertiary-care university hospital in Eskişehir, Türkiye, between 1 February 2017 and 30 May 2017. Eskişehir is among Türkiye’s socioeconomically developed provinces, with a total population of 921,630, 3 public universities with approximately 70,000 students and a single medical faculty [25]. With its 1000-bed capacity, the medical faculty serves not only Eskişehir but also patients from surrounding provinces. In the emergency department, resident physicians and specialists work in shifts [26].

**Figure 1:**
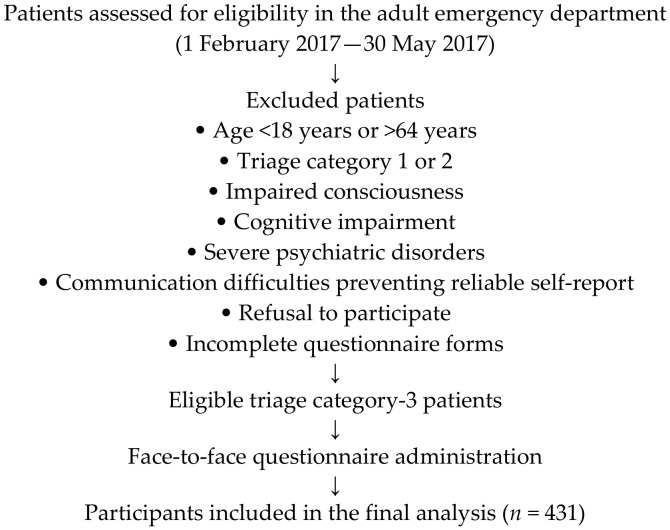
Flowchart of patient selection and inclusion process.

Patients aged 18–64 years who were classified as triage category 3 according to emergency department triage assessment, were clinically stable, and were able to communicate independently were included in the study. Data were obtained directly from the patients themselves; proxy respondents such as relatives or caregivers were not included. Triage category 3 includes clinically stable patients with non–life-threatening conditions who can safely wait for evaluation and are capable of providing reliable self-reported data. Patients classified as triage categories 1 and 2 were excluded because these patients require immediate or urgent life-saving interventions, making administration of a health literacy questionnaire ethically inappropriate and methodologically unreliable due to acute distress, altered consciousness, severe pain, or hemodynamic instability. In addition, patients with impaired consciousness, cognitive impairment, severe psychiatric disorders, or communication difficulties preventing reliable self-report were excluded from the study. Patients aged 65 years and older were also excluded in order to minimize potential bias related to cognitive impairment, impaired mental functioning, difficulties in reliable self-reporting, and the high burden of chronic diseases that may affect both health literacy levels and emergency department utilization patterns. The patient selection and inclusion process are summarized in Figure 1.

The minimum required sample size was calculated as at least 384 participants, assuming an inadequate health literacy prevalence of 50%, a 95% confidence level, and a 5% margin of error. To minimize potential non-response and incomplete data, recruitment continued beyond the minimum required sample size. After the necessary examinations and tests were completed, triage category-3 patients were informed about the purpose and scope of the study. Patients who agreed to participate completed the questionnaire through face-to-face interviews conducted by resident physicians. Before data collection, all research assistants received standardized training regarding questionnaire administration in order to minimize inter-observer bias. No specific time interval was defined for data collection; data were collected during 24 h shifts when patient density was appropriate. A total of 431 participants were included in the final analysis.

The data for this study were collected using a two-part structured questionnaire developed by the researchers based on a review of the relevant literature [27,28]. The first part of the questionnaire consisted of descriptive questions regarding sociodemographic characteristics and emergency department utilization patterns. The second part included the widely used Chew short questions. Due to the high patient flow and time-sensitive nature of the emergency department setting, a brief and practical screening tool was preferred instead of longer multidimensional health literacy instruments. The Chew screening questions were used as a brief screening tool to identify inadequate health literacy rather than as a comprehensive measure of critical health literacy. In this study, the Turkish version of the four-item Chew Health Literacy Screening Questions, adapted and validated by Eyüboğlu et al., was used to assess inadequate health literacy.

This quantitative cross-sectional study used structured questionnaires administered through face-to-face interviews. The questionnaires were completed directly by the participants themselves, while trained resident physicians provided assistance when necessary. Before data collection began, all resident physicians involved in the study received standardized training regarding questionnaire administration and data collection procedures in order to minimize inter-observer bias. Data collection was performed during 24 h emergency department shifts, including daytime, evening, and night shifts, depending on patient density and workflow suitability. Therefore, patients presenting at different times of the day were included in the study in order to reduce potential time-related selection bias. Written informed consent was obtained from all participants prior to participation. Ethical approval was obtained from the Eskişehir Osmangazi University Non-Interventional Clinical Research Ethics Committee (approval number: 11, date: 13 February 2017), and necessary permissions were obtained from the hospital administration.

The CHEW offers a practical advantage due to its brevity, speed, and ease of application, while also demonstrating validity and reliability in Turkish. Chew’s short questions are primarily designed as a brief screening tool for functional health literacy rather than a comprehensive multidimensional assessment of health literacy domains such as communicative, critical, or digital health literacy. Chew screening questions:

(1) How often do you have someone help you read hospital materials?
(2) How confident are you filling out medical forms by yourself?
(3) How often do you have problems learning about your medical condition because of difficulty understanding written information?
(4) How often do you have problems understanding what is told to you about your medical condition?

The Turkish version of the Chew scale is a 5-point Likert-type scale in which a score of 1 represents “never” and a score of 5 represents “always,” with higher scores indicating lower health literacy levels. In accordance with the Turkish validation study of Chew’s short questions, participants who reported any degree of difficulty in at least one item (responses of “rarely,” “sometimes,” “often,” or “always”) were classified as having inadequate health literacy, whereas participants who responded “never” to all items were classified as having adequate health literacy [27,28].

Data analysis was conducted using IBM SPSS version 27.0. Descriptive statistics, including frequency, percentage, and mean, were used to summarize the characteristics of the study group. The Chi-square test was applied for data analysis, with statistical significance set at *p* < 0.05. To determine the independent factors associated with inadequate health literacy, a multivariable logistic regression analysis was performed. Variables considered clinically and theoretically relevant to health literacy were included in the multivariable logistic regression analysis regardless of their statistical significance in univariate analyses. The regression model was constructed to identify factors independently associated with inadequate health literacy and included age, sex, educational level, perceived income level, employment status, number of emergency department visits, and repeated emergency department admissions within the previous six months as potential confounding variables. Adjusted odds ratios (ORs) with 95% confidence intervals (CIs) were calculated.

## 3. Results

The patient selection and inclusion process are presented in Figure 1.

The study population consisted of 250 females (58.0%) and 181 males (42.0%) participants. Participants were between 18 and 64 years of age, with a mean (SD) age of 29.6 (10.8) years and a median age of 26.0 years. Overall, 197 participants (45.7%) were classified as having inadequate health literacy. The frequency of inadequate health literacy was significantly higher among participants aged ≥40 years compared with younger participants (*p* = 0.030). Likewise, participants with an education level of ≤8 years had significantly higher rates of inadequate health literacy compared with those with higher educational attainment (*p* = 0.022). In contrast, the frequency of inadequate health literacy decreased as perceived income level increased (*p* = 0.004). Table 1 presents the comparison of participants’ demographic and background characteristics according to health literacy status.

Variables considered clinically and theoretically relevant to health literacy, including age group, sex, educational status, perceived income level, employment status, number of emergency department visits, and repeated emergency department admission within the previous six months, were included in the multivariable logistic regression model. The results demonstrated that perceived income level was independently associated with health literacy status. Compared with participants reporting poor income status, those with medium income levels had 1.83 times higher odds of adequate health literacy (OR = 1.830, 95% CI: 1.115–3.003, *p* = 0.017), while participants with good income levels had 2.50 times higher odds of adequate health literacy (OR = 2.502, 95% CI: 1.205–5.193, *p* = 0.014). In contrast, age group and educational status were not independently associated with health literacy after adjustment for potential confounding variables (*p* > 0.05). The results of the logistic regression analysis are presented in Table 2.

A total of 169 participants (39.2%) reported repeated emergency department visits for the same complaint, while 243 participants (56.4%) reported at least one emergency department visit for a different complaint within the previous six months. These categories were not mutually exclusive, and some participants reported both repeated visits for the same complaint and visits for different complaints during the same period. Table 3 presents the characteristics of emergency department utilization. Among patients with repeated visits for the same complaint, the most commonly reported reason for revisiting was recurrence of similar symptoms. Because multiple responses were allowed, the total number of responses presented in Table 4 exceeded the number of participants with repeated visits. Table 4 summarizes the reasons for repeated visits.

## 4. Discussion

Rising ED crowding and increasing patient volume may negatively affect patient safety and quality of care. Inadequate health literacy—highly prevalent at the population level in Türkiye—has been associated with greater ED utilization and may contribute to potentially avoidable or non-urgent ED visits as one of multiple determinants [29,30]. This study aimed to assess the HL levels of patients of the emergency department of a university hospital in a city.

One of the notable findings of this study was the high prevalence of inadequate health literacy despite the relatively young age profile of the sample and the study being conducted in a socioeconomically developed province with a large university population. In this study, 45.7% of patients were found to have problematic HL. This result is consistent with other community-based studies conducted in Turkey over the years. A study reported that 64.6% of the population had “inadequate” (24.5%) or “problematic” (40.1%) HL, whereas another study found 30.9% with inadequate and 38% with inadequate HL levels [31,32]. Similarly, a study conducted in Erzurum, Türkiye, found that 82.8% of participants had inadequate or inadequate HL [33]. Although the current study expected HL levels to be lower in emergency department patients, it did not confirm this; however, the study conducted in Erzurum did confirm this. This discrepancy could be because of regional socio-economic differences or the fact that our participant group was limited to individuals aged 18–64 years. In contrast, Griffey et al. [34] reported varying results for inadequate HL using three different assessment tools: 23.9% (S-TOFHLA), 48.5% (REALM-R), and 76.7% (NVS). In the study conducted by Carpenter et al. [22], three different HL scales were used, and the frequencies of inadequate HL levels varied across the three scales. This variation may be due to the lack of a standardized measurement tool for determining the HL level in emergency departments and the differences in the evaluation characteristics of the measurement tools used.

Systematic reviews on aging and HL have consistently demonstrated a strong association between older age and poor HL [35,36]. The findings of this study align with the literature, indicating that the frequency of inadequate HL was higher in older age groups. Studies conducted in France showed that older age is a risk factor for inadequate HL, whereas studies in Germany and the US did not identify an association between age and HL levels [24,37,38]. In fact, some studies have even shown a positive correlation between age and HL levels [39,40]. Several factors may contribute to the decline in HL levels among older individuals, including reduced cognitive function such as memory loss, difficulty in understanding and learning new information, physical impairments such as hearing and vision loss, psychosocial factors, and socio-economic status. Given the variability of these factors in older adults, future research should focus on individual differences in these aspects.

The reasons behind the differences in HL levels between genders are not fully understood. Access to healthcare services, usage patterns, and help-seeking behaviors may vary according to gender [41]. Some studies have reported that women tend to have lower HL levels than men [31,32,42], whereas others found no association between gender and HL levels [33,43]. A few studies even suggest that women may have higher HL levels [44,45]. This study found no significant relationship between gender and HL levels. Factors such as the sociocultural context, women’s participation in educational activities, and their roles in the workforce can all influence literacy skills, thus contributing to varied results. Therefore, further large-scale studies that control the confounding factors and standardize participant groups are required to better understand these dynamics. In this study, individuals with lower education and income levels had significantly lower HL levels. This finding is consistent with the results of community-based studies in Türkiye [31,32]. However, a study by Yılmaz et al. [33] in Türkiye found no relationship between income and HL levels, although individuals with lower education levels did have significantly lower HL levels. In contrast, a study conducted in France found no relationship between education, income, and HL levels [38]. Similarly, Ginde et al. [10] demonstrated a positive relationship between income, education level, and HL in their study. Education and income are fundamental determinants of socio-economic status. As such, higher socio-economic status and better literacy skills can positively influence access to accurate health information, processing of that information, and decision-making. However, it is important to note that while HL is related to basic literacy, it is not entirely synonymous with it. HL is a much more complex concept. In the multivariable analysis, perceived income level remained independently associated with health literacy, whereas the associations with age and educational status were no longer statistically significant after adjustment for potential confounding factors. This finding may suggest that socioeconomic conditions and perceived financial resources have a stronger influence on functional health literacy in this study population than demographic characteristics alone. Individuals with lower perceived income may experience greater barriers in accessing, understanding, and using health-related information and healthcare services.

In this study, approximately four out of every ten patients reported repeated emergency department visits within the last six months for the same complaint. In addition, a considerable proportion of patients also reported emergency department visits for different complaints during the same period. These findings reflect overlapping patterns of emergency department utilization rather than mutually exclusive categories. However, no statistically significant relationship was found between health literacy levels and emergency department visit characteristics. Similarly, Ginde et al.’s cross-sectional study also did not find a relationship between HL levels and emergency department usage patterns. In contrast, several longitudinal studies have reported that inadequate HL levels are associated with an increased risk and c visits [4,11,46]. In this study, the most common reasons for repeated visits were recurrence of symptoms despite medication and difficulties in accessing outpatient services. Previous studies have suggested that system-related factors, including limited timely access to primary care services, difficulty obtaining appointments, physician workload, and the perceived advantages of emergency departments such as 24/7 availability and faster diagnostic evaluation, may contribute to increased emergency department utilization in Türkiye [47,48]. According to the most recent health statistics in Türkiye, there were 973,519,087 healthcare visits in 2023, of which 56.7% were to secondary or tertiary healthcare facilities. Additionally, the number of patient visits per physician was reported as 11.4, placing Türkiye among the leading OECD countries in terms of physician workload despite having relatively low physician numbers per capita [49,50]. Furthermore, the absence of a statistically significant association in this study may partly be explained by the restriction of the sample to triage category-3 patients and by the potential influence of organizational and healthcare access factors that may play a more dominant role than individual health literacy differences in emergency department utilization patterns.

### Limitations

This study has several limitations. First, the cross-sectional design provides a snapshot in time and does not allow causal inference or conclusions about temporal relationships. Second, health literacy was assessed using a brief self-report measure, which may be prone to reporting and social desirability bias and may not fully capture the multidimensional nature of health literacy. Third, the single-center setting and sample-specific characteristics may limit the generalizability of our findings. In particular, Eskişehir has a large student population; therefore, the age distribution of emergency department attendees in our setting may not fully reflect the demographic profile of the broader local community, potentially limiting external validity. Fourth, restricting inclusion to triage category 3 patients further reduces representativeness for the overall emergency department population. Fifth, unmeasured factors (e.g., access to primary care, socioeconomic status, and comorbidity) may have influenced the observed findings. Sixth, although multidimensional instruments such as the HLS-EU-Q provide a more comprehensive assessment of health literacy, the Turkish version of Chew’s short questions was preferred in this study because of its practicality and feasibility in a busy emergency department setting. Therefore, the use of a brief screening instrument may not have fully reflected the multidimensional nature of health literacy and may have contributed to the lack of statistically significant associations with emergency department utilization characteristics. Seventh, another limitation of this study is the interval between data collection and manuscript submission. Since the data were collected in 2017, the findings should be interpreted within the context of the pre-pandemic period. Changes in healthcare utilization patterns, access to health information, digital health communication, and emergency department use after the COVID-19 pandemic may limit the direct applicability of the findings to current patient populations.

## 5. Conclusions

In conclusion, our study found that four out of every ten individuals had inadequate HL levels and the frequency of repeated emergency department visits was notably high. No statistically significant association was found between emergency department usage characteristics and health literacy levels in the present sample. However, further large-scale longitudinal studies with adjusted analyses are needed to better clarify this relationship.

## Figures and Tables

**Table 1:** Comparison of demographic and background characteristics according to health literacy status.

Variables		*n* (%)	HL Level		Statistical Analysis Chi-Square; *p*
			Adequate *n* (%)	Inadequate *n* (%)	
Age Group	≤29	264 (61.3)	149 (56.4)	115 (43.6)	**7.009; 0.030**
	30–39	91 (21.1)	54 (59.3)	37 (40.7)	
	**≥40**	**76 (17.6)**	**31 (40.8)**	**45 (59.2)**	
Gender	Female	250 (58.0)	143 (57.2)	107 (42.8)	2.028; 0.154
	Male	181 (42.0)	91 (50.3)	90 (49.7)	
Education Level	**≤8 years**	**79 (18.3)**	**32 (40.5)**	**47 (59.5)**	**7.590; 0.022**
	High School	106 (24.6)	59 (55.7)	47 (44.3)	
	University	246 (57.1)	143 (58.1)	103 (41.9)	
Marital Status	Married	183 (42.5)	92 (50.3)	91 (49.7)	2.070; 0.150
	Unmarried	248 (57.5)	142 (57.3)	106 (42.7)	
Income-generating Work Status	Unemployed	169 (39.2)	141 (53.8)	121 (46.2)	0.061; 0.805
	Employed	262 (60.8)	93 (55.0)	76 (45.0)	
Perceived Income Level	**Poor**	**93 (21.6)**	**37 (39.8)**	**56 (60.2)**	**11.162; 0.004**
	**Medium**	**284 (65.9)**	**162 (57.0)**	**122 (43.0)**	
	**Good**	**54 (12.5)**	**35 (64.8)**	**19 (35.2)**	
Household Size	≤3	142 (32.9)	80 (56.3)	62 (43.7)	0.357; 0.550
	≥4	289 (67.1)	154 (53.3)	135 (46.7)	
Place of Residence	Urban Center	359 (83.3)	195 (54.3)	164 (45.7)	0.001; 0.981
	Periphery	72 (16.7)	39 (54.2)	33 (45.8)	
Distance Between Residence and Health Facility	By car	158 (36.7)	80 (50.6)	78 (49.4)	1.346; 0.246
	By walking	273 (63.3)	154 (56.4)	119 (43.6)	
Physician-diagnosed Chronic Disease History	Yes	70 (16.2)	43 (61.4)	27 (38.6)	1.715; 0.190
	No	361 (83.8)	191 (52.9)	170 (47.1)	
Total		431 (100.0)	234 (54.3)	197 (45.7)	

**Table 2:** Multivariable logistic regression analysis of factors associated with adequate health literacy.

Variable		OR (Exp(B))	*p*	95% CI
**Age group**	≤29	1	-	-
	30-39	1.151	0.617	0.664–1.995
	≥40	0.603	0.097	0.331–1.096
**Educational status**	≤8 years	1	-	-
	High School	1.491	0.219	0.788–2.822
	University	1.440	0.247	0.777–2.670
**Income level**	**Poor**	1	-	-
	**Medium**	1.839	0.018	1.109–3.048
	**Good**	2.548	0.014	1.210–5.363

**Table 3:** Emergency department utilization characteristics of the participants.

Variables			*n* (%)	HL Level		Statistical Analysis Chi-Square; *p*
				Adequate *n* (%)	Inadequate *n* (%)	
First Visit for Current Complaint	No		169 (39.2)	85 (50.3)	84 (49.7)	1.789; 0.181
	Yes		262 (60.8)	149 (56.9)	113 (43.1)	
Visited Emergency Department in Last 6 Months (Other than Current Visit)	No		188 (43.6)	104 (55.3)	84 (44.7)	0.142; 0.707
	Yes		243 (56.4)	130 (53.5)	113 (46.5)	
Total		431 (100.0)	234 (54.3)	197 (45.7)		

**Table 4:** Reasons for repeat emergency department visits.

Reasons for Repeat Emergency Department Visits		*n* * (%)
Recurrent complaint	Inability to access outpatient services/late arrival	22 (10.9)
	Symptoms persisting despite medication	60 (29.8)
	Resumption of similar complaints	100 (49.8)
	Inability to access/understand treatment	14 (7.0)
	Dissatisfaction	5 (2.5)
Total		201 (100.0)

* Multiple responses were allowed. Numbers and percentages are based on the indicated responses.

## Data Availability

The datasets generated and/or analyzed during the current study are available from the corresponding author on reasonable request.

## References

[1] Nutbeam D., Muscat D.M. (2021). Health Promotion Glossary 2021. Health Promot. Int..

[2] Ad Hoc Committee on Health Literacy for the Council on Scientific Affairs, American Medical Association (1999). Health literacy: Report of the Council on Scientific Affairs. JAMA.

[3] Baker D.W., Gazmararian J.A., Williams M.V., Scott T., Parker R.M., Green D., Ren J., Peel J. (2002). Functional health literacy and the risk of hospital admission among Medicare managed care enrollees. Am. J. Public Health.

[4] Baker D.W., Gazmararian J.A., Williams M.V., Scott T., Parker R.M., Green D., Ren J., Peel J. (2004). Health literacy and use of outpatient physician services by Medicare managed care enrollees. J. Gen. Intern. Med..

[5] Shahid R., Shoker M., Chu L.M., Frehlick R., Ward H., Pahwa P. (2022). Impact of low health literacy on patients’ health outcomes: A multicenter cohort study. BMC Health Serv. Res..

[6] Cho Y.I., Lee S.-Y.D., Arozullah A.M., Crittenden K.S. (2008). Effects of health literacy on health status and health service utilization among the elderly. Soc. Sci. Med..

[7] DeWalt D.A., Berkman N.D., Sheridan S., Lohr K.N., Pignone M.P. (2004). Literacy and health outcomes: A systematic review of the literature. J. Gen. Intern. Med..

[8] DeWalt D.A., Dilling M.H., Rosenthal M.S., Pignone M.P. (2007). Low parental literacy is associated with worse asthma care measures in children. Acad. Pediatr..

[9] Fortenberry J.D., McFarlane M.M., Hennessy M., Bull S.S., Grimley D.M., Lawrence J.S., Stoner B.P., VanDevanter N. (2001). Relation of health literacy to gonorrhea-related care. Sex. Transm. Infect..

[10] Ginde A.A., Clark S., Goldstein J.N., Camargo C.A. (2008). Demographic disparities in numeracy among emergency department patients: Evidencefrom two multicenter studies. Patient Educ. Couns..

[11] Ginde A.A., Weiner S.G., Pallin D.J., Camargo C.A. (2008). Multicenter study of inadequate health literacy in emergency department patients. Acad. Emerg. Med..

[12] Howard D.H., Gazmararian J., Parker R.M. (2005). The impact of low health literacy on the medical costs of Medicare managed care enrollees. Am. J. Med..

[13] Lindau S.T., Tomori C., Lyons T., Langseth L., Bennett C.L., Garcia P. (2002). The association of health literacy with cervical cancer prevention knowledge and health behaviors in a multiethnic cohort of women. Am. J. Obstet. Gynecol..

[14] Mancuso C.A., Rincon M. (2006). Impact of health literacy on longitudinal asthma outcomes. J. Gen. Intern. Med..

[15] Omachi T.A., Sarkar U., Yelin E.H., Blanc P.D., Katz P.P. (2013). Lower health literacy is associated with poorer health status and outcomes in chronic obstructive pulmonary disease. J. Gen. Intern. Med..

[16] Schillinger D., Grumbach K., Piette J., Wang F., Osmond D., Daher C., Palacios J., Sullivan G.D., Bindman A.B. (2002). Association of health literacy with diabetes outcomes. JAMA.

[17] Scott T.L., Gazmararian J.A., Williams M.V., Baker D.W. (2002). Health literacy and preventive health care use among Medicare enrollees in a managed care organization. Med. Care.

[18] Sudore R.L., Yaffe K., Satterfield S., Harris T.B., Mehta K.M., Simonsick E.M., Newman A.B., Rosano C., Rooks R., Rubin S.M. (2006). İnadequate literacy and mortality in the elderly: The Health, Aging, and Body Composition Study. J. Gen. Intern. Med..

[19] Wolf M.S., Gazmararian J.A., Baker D.W. (2005). Health literacy and functional health status among older adults. Arch. Intern. Med..

[20] American College of Emergency Physicians Definition of an Emergency Service [Internet]. ACEP. https://www.acep.org/patient-care/policy-statements/definition-of-an-emergency-service.

[21] Griffey R.T., Kennedy S.K., McGownan L., Goodman M., Kaphingst K.A. (2014). Is low health literacy associated with increased emergency department utilization and recidivism?. Acad. Emerg. Med..

[22] Carpenter C.R., A Kaphingst K., Goodman M.S., Lin M.J., Melson A.T., Griffey R.T. (2014). Feasibility and diagnostic accuracy of brief health literacy and numeracy screening instruments in an urban emergency department. Acad. Emerg. Med..

[23] Herndon J.B., Chaney M., Carden D. (2011). Health literacy and emergency department outcomes: A systematic review. Ann. Emerg. Med..

[24] Balakrishnan M.P., Herndon J.B., Zhang J., Payton T., Shuster J., Carden D.L. (2017). The association of health literacy with preventable emergency department visits: A cross-sectional study. Acad. Emerg. Med..

[25] Türkiye İstatistik Kurumu (TÜİK). Medas Veri Portalı [Internet]. https://biruni.tuik.gov.tr/medas.

[26] Eskişehir Osmangazi Üniversitesi Tıp Fakültesi Fakülte Tarihçesi [Internet]. https://tip.ogu.edu.tr/Sayfa/Index/1176/tarihce.

[27] Eyüboğlu E., Schulz P.J. (2016). Validation of Turkish health literacy measures. Health Promot. Int..

[28] Chew L.D., Bradley K.A., Boyko E.J. (2004). Brief questions to identify patients with inadequate health literacy. Fam. Med..

[29] Özdemir S., Akça H.Ş., Algın A., Kokulu K. (2020). Health literacy in the emergency department: A cross-sectional descriptive study. Eurasian J. Emerg. Med..

[30] Çalık M., Kolaç N. (2025). Yetişkin hastaların sağlık okuryazarlık düzeyine göre acil servisi kullanma durumları ve etkileyen faktörler. Halk Sağ. Hem. Der..

[31] Tanriöver M.D., Yildirim H.H., Ready F.N.D., Çakir B., Akalin H.E. (2014). Türkiye Sağlık Okuryazarlığı Araştırması.

[32] Özkan S., Tüzün H., Dikmen A.U., Aksakal N.B., Çalışkan D., Taşçı Ö., Güneş S.C. (2021). The relationship between health literacy level and media used as a source of health-related information. Health Lit. Res. Pract..

[33] Yılmaz S., Gür S.T.A., Daharlı E. (2021). Examination of the relationship between emergency department usage and health literacy: A cross-sectional study. J. Anatol. Med. Res..

[34] Griffey R.T., Melson A.T., Lin M.J., Carpenter C.R., Goodman M.S., Kaphingst K.A. (2014). Does numeracy correlate with measures of health literacy in the emergency department?. Acad. Emerg. Med..

[35] Lima A.C.P., Maximiano-Barreto M.A., Martins T.C.R., Luchesi B.M. (2024). Factors associated with poor health literacy in older adults: Asystematic review. Geriatr. Nurs..

[36] Marshall N., Butler M., Lambert V., Timon C.M., Joyce D., Warters A. (2025). Health literacy interventions and health literacy-related outcomes for older adults: Asystematic review. BMC Health Serv. Res..

[37] Strauß A., Zimmermann T., Schäfer I., Scherer M. (2022). Health literacy and commitment to a general practitioner in low-acuity patients ofemergency departments. Z. Evid. Fortbild. Qual. Gesundheitswes..

[38] Marie P., Romain-Scelle N., Potinet V., Schott A.M., Douplat M. (2024). Assessment of health literacy in a French emergency department. BMC Health Serv. Res..

[39] Tiller D., Herzog B., Kluttig A., Haerting J. (2015). Health literacy in an urban elderly East-German population. BMC Public Health.

[40] Nakayama K., Osaka W., Togari T., Ishikawa H., Yonekura Y., Sekido A., Matsumoto M. (2015). Comprehensive health literacy in Japan is lower than in Europe. BMC Public Health.

[41] Sun S., Lu J., Wang Y., Wang Y., Wu L., Zhu S., Zheng X., Lu X., Xu H. (2022). Gender differences in factors associated with the health literacy of hospitalized older patients with chronic diseases: A cross-sectional study. Front. Public Health.

[42] Mashi A.H., Aleid D., Almutairi S., Khattab F., AlMuqawed A.N., Khan S., AlBanyan N., Brema I., AlJohani N.J. (2019). The association of health literacy with glycemic control in Saudi patients with type 2 diabetes. Saudi Med. J..

[43] von der Warth R., Körner M., Farin-Glattacker E. (2024). Health literacy of trans and gender diverse individuals: A cross-sectional survey in Germany. 2024. BMC Public Health.

[44] Clouston S.A.P., Manganello J.A., Richards M. (2017). A life course approach to health literacy. Age Ageing.

[45] Aljahany M., Doumi R., Alhuthail R.A., Alshangiti H.Y., Alsugair R.A., Aldokhail L.S., Aljohani L.H., Alqasimi N.A., Alotaibi E.M., Alaradi L.M. (2024). Public health literacy and emergency department utilization. Risk Manag. Health. Policy.

[46] Baker D.W., Parker R.M., Williams M.V., Clark W.S. (1998). Health literacy and the risk of hospital admission. J. Gen. Intern. Med..

[47] Vogel J.A., Rising K.L., Jones J., Bowden M.L., Ginde A.A., Havranek E.P. (2019). Reasons patients choose the emergency department over primary care. J. Gen. Intern. Med..

[48] Wu N., Woloski J.R. (2024). Emergency department versus primary care use: A patient perspective. PRiMER.

[49] Republic of Türkiye Ministry of Health Health Statistics Yearbook 2023 [Internet]. https://www.saglik.gov.tr.

[50] Organisation for Economic Co-operation and Development (OECD) OECD Data Explorer [Internet]. https://data-explorer.oecd.org.

